# Learning Style Profiles of Second-Year MBBS Students in a Medical College Situated in Remote Islands of India

**DOI:** 10.7759/cureus.98491

**Published:** 2025-12-04

**Authors:** Ann George, K Chandrahasan, Senkadhirdasan Dakshinamurthy

**Affiliations:** 1 Pharmacology, Andaman and Nicobar Islands Institute of Medical Sciences, Pondicherry University, Sri Vijaya Puram, IND; 2 Community Medicine, Mahatma Gandhi Medical College and Research Institute, Sri Balaji Vidyapeeth University, Pondicherry, IND

**Keywords:** academic performance, learning style, medical students, study hours, study method, study pattern

## Abstract

Context

Though extensively studied, the relationship between learning styles and academic performance in medical education remains inconclusive. The present study looks into this relationship among second-year students in a medical college in Andaman and Nicobar Islands in India, where the cultural diversity of students may add to the diversity of their learning styles and strategies.

Aims

The aims of this study are: (i) to evaluate the students' learning style preference on four dimensions based on the Felder-Silverman model; (ii) to assess the association between learning style preference on four dimensions and gender, study hours, study method and study pattern; and (iii) to determine the association between learning style preference on four dimensions, gender, study hours, study method, study pattern and academic performance.

Settings and design

This is a cross-sectional questionnaire-based study done on the Bachelor of Medicine and Bachelor of Surgery (MBBS) students of the Andaman and Nicobar Islands Institute of Medical Sciences (ANIIMS) who attend second-year classes.

Materials and methods

After obtaining approval from Institutional Research Ethics Committee, 154 medical students who enrolled in the second year during the study period were given an information sheet describing the project. Those who consented to participate were requested to complete the Index of Learning Styles (ILS) Questionnaire and a semi-structured questionnaire. The first internal assessment marks scored in Pharmacology were taken as the academic score.

Statistical analysis

Data was entered in Microsoft Excel and statistical analysis was conducted. First, descriptive statistics were derived. Chi-square test, one-way analysis of variance (ANOVA) and post hoc analysis were performed.

Results

Gender distribution showed higher proportion of female students than male students (107 (69.5%) vs 47(30.5%)). In the study, 94 (61%) students studied one to three hours per day and 91 (59%) students took to an individual study. According to the results, 85 (55%) students followed a regular pattern of studying throughout the year while 69 (45%) students studied more just before the exams. In the ‘Processing’ dimension of ILS, 104 (67.5%) students’ preference is fairly well balanced on the axis. In the ‘Perception’ dimension, 79 (51%) students’ preference is fairly well balanced on the axis. In the ‘Input Dimension’, 72 (47%), students have moderate preference to the visual axis. In the ‘Comprehension’ dimension, 100 (65%) students’ preference is fairly well balanced on the axis. A statistically significant association was observed between study hours and sensing/intuitive preference (p=0.03). Significant associations were seen in case of gender (p=0.006) and study pattern (p=0.039), and the sequential-global axis. Male students showed a higher global learning preference, while irregular study patterns were associated with intuitive styles. Students following a year-round study pattern scored higher with p=0.006. Male students scored slightly better than female students (p=0.03).

Conclusion

This study concludes that consistent study habit appears to be more influential on academic performance than inherent learning styles.

## Introduction

Learning is a very complex process that is influenced by several factors such as intelligence, motivation, proper environment, domestic factors, community, school quality, and teacher quality. Similarly, the learning style of learners is another factor contributing to their learning [1]. Learners’ learning styles have been defined as a multistep process that requires information intake, processing, preserving, and recall and are used to acquire knowledge or a skill [2]. Keefe defines learning styles as a combination of cognitive, emotional, and physiological features that serve as relatively stable indicators of how learners perceive, interact with, and respond to the learning environment [3]. The efforts to understand learning and thinking styles and to learn to use them flexibly require the identification of an individual’s preferred style of learning and thinking. Styles like abilities are partly developed due to environmental conditions and are modifiable [4].

The primary aim of teaching is to facilitate the learning process [5]. Understanding the learning behavior of students is considered to be a part of this process [6]. Therefore, the concept of learning styles has become a popular topic in recent literature, with many theories about learning styles put forward to better understand the dynamic process of learning like the Kolb’s model, Felder and Silverman’s model, and Gregorc’s mind styles model [7,8]. However, it’s important to note that the relationship between learning styles and academic performance is not simple or direct.

Regardless of the context or modality, a common theme emerges in the literature: the importance of tailoring instruction to the student’s learning style. A body of research suggests that by aligning the teaching strategy with the student’s preferred learning style, one can enhance student engagement, understanding of material, and ultimately, academic performance, while empirical studies have shown limited support for this approach [9-11].

Specifically, research indicates that many students do not study in ways that align with their self-reported learning styles, and that matching instruction to these styles does not necessarily improve performance [12]. Each of them will have variations in individual constraints, experiences, and preferences [13]. The diversity in educational backgrounds, cultural influences, ethnic origins, and gender identities among the contemporary Bachelor of Medicine and Bachelor of Surgery (MBBS) students presents unique challenges and opportunities for educators.

Felder and Silverman (1988) developed the Index of Learning Styles (ILS) [14]. ILS is a comprehensive indicator of students' learning style. This is a questionnaire-based assessment. Among its four dimensions, the 'Processing' dimension includes the active-reflective axis, which finds whether students prefer to engage with others during the learning process or prefer to reflect individually when acquiring new information [15]. Under the 'Perception' dimension, students are divided along the intuitive-sensing axis: intuitive learners favor abstraction and tend to avoid memorization, whereas sensing learners excel when dealing with real-world facts and follow procedural learning, which includes memorization [16]. For the 'Input' dimension, students are classified as visual learners who prefer diagrams, drawings, and schemes or as verbal learners who favor written or spoken information. The 'Comprehension' dimension includes the sequential learners who need to learn in small, ordered steps, and global learners who prefer a holistic view of the phenomenon, which allows them to solve problems innovatively and quickly. This model has several advantages over other approaches, including the possibility of assessing multiple learning styles, brevity, ease of administration, free access, and appropriate psychometric characteristics of the ILS [17-19].

Some students do not show a clear preference for any single axis and can adapt to different learning styles with ease [16,20]. We must identify the profile of medical students in order to know about distress and competency and its impact on academic performance, dropout rates, and professional development [21]. In addition, by consciously choosing to engage all the learning styles, facilitators can help students adapt to learning by non-preferred methods. While it is often assumed that these diverse backgrounds necessitate a personalized approach to learning, it is crucial to recognize that individual learning preferences may not always align with optimal learning strategies [22]. Training in non-preferred learning styles can be useful to students in their future professional lives, where the ability to learn and gather information in multiple ways can be critical to success. Effective education should focus on evidence-based instructional methods that cater to a wide range of learners, while also promoting critical thinking, adaptability, and lifelong learning skills.

The relationship between learning styles and academic performance in medical education has been the subject of numerous studies, with varying results [23-27]. The inconsistent results in previous studies and lack of studies among the students in the present institution motivated the researchers to take up the present topic to characterize the learning styles of medical students using the ILS of Felder and Silverman.

## Materials and methods

This is a cross-sectional questionnaire-based study. The study involves only one group, i.e., all second-year MBBS students of Andaman Nicobar Institute of Medical Sciences, Sri Vijaya Puram, India. A convenience sampling method was used and all students who attended Pharmacology during the study period were included in the study. After obtaining approval from the Institutional Ethics Committee, 158 second-year MBBS students were given an information sheet describing the project. Those students who didn’t give informed consent, didn’t complete the questionnaires or who were absent at the time of data collection were excluded from the study. Finally, 154 (97.5%) second-year MBBS students (2023 batch) who satisfied the inclusion criteria signed the informed consent form and completed the ILS questionnaire and a semi-structured questionnaire. Using ILS, the learning styles are categorized into four bipolar dimensions: the way an individual processes information (active/ reflective), the preferred way of information perception (sensing/intuitive), the preferred sensory input channel (visual/ verbal), and the progression towards understanding (sequential/global). There are 11 questions for each of the four dimensions. The response to each question is binary and they are totaled to find the preference. The score will have a number and an alphabet. The number will indicate the degree of preference and the alphabet will categorize between the two options in the dimension. The semi-structured questionnaire collected details like study hours, study method, study pattern, etc. Marks obtained in the first internal assessment in Pharmacology were taken as the academic score of the student in percentage.

Data was entered in Microsoft Excel (Microsoft, Redmond, WA) and statistical analyses were done. First, descriptive statistics like frequency, mean and standard deviation (SD) were derived. Chi-square test was used to find any association between categorical variables (like gender, study hours, study method, study pattern) and score in each dimension of the learning style profile. Any association between categorical variables (like gender, study hours, study method, study pattern, score in each dimension of learning style profile) and academic performance (quantitative variable) was looked into using one-way analysis of variance (ANOVA). Post hoc analysis was done for significant associations.

## Results

Gender distribution showed a higher proportion of female students (69.5%) than male students (30.5%). The majority of the students (61%) studied one to three hours per day. Most of the students (59%) followed the method of individual study, whereas 55% of students had a regular pattern of studying throughout the year. But 45% of students studied more just before exams. In the ‘Processing’ dimension of ILS, 67.5% of students’ preference is fairly well balanced on the active-reflective axis. In the ‘Perception’ dimension, 51% of students’ preference is fairly well balanced on the intuitive-sensing axis. In the ‘Input Dimension’, 47% of the students have moderately prefer the visual axis to verbal axis. In the ‘Comprehension’ dimension, 65% of students’ preference is fairly well balanced on the sequential-global axis (Table 1).

**Table 1:** Frequency Distribution Table

Variable	Category	Count (n)	Column (%)
Gender	Male (1)	47	30.5%
	Female (2)	107	69.5%
Study Hours	<1 hour (1)	33	21.4%
	1-3 hours (2)	94	61.0%
	>3 hours (3)	27	17.5%
Study Method	Group study (1)	6	3.9%
	Individual (2)	91	59.1%
	Both methods (3)	57	37.0%
Study Pattern	Throughout year (1)	85	55.2%
	Irregular (2)	69	44.8%
'Processing' dimension	1=Fairly well balanced	104	67.5%
	2=Moderate preference active	22	14.3%
	3=Strong preference active	3	1.9%
	4=Moderate preference reflective	23	14.9%
	5=Strong preference reflective	2	1.3%
'Perception' dimension	1=Fairly well balanced	79	51.3%
	2=Moderate preference sensing	50	32.5%
	3=Strong preference sensing	7	4.5%
	4=Moderate preference intuitive	17	11.0%
	5=Strong preference intuitive	1	0.6%
'Input' dimension	1=Fairly well balanced	45	29.2%
	2=Moderate preference visual	72	46.8%
	3=Strong preference visual	34	22.1%
	4=Moderate preference verbal	3	1.9%
'Comprehension' dimension	1=Fairly well balanced	100	64.9%
	2=Moderate preference sequential	30	19.5%
	3=Strong preference sequential	3	1.9%
	4=Moderate preference global	19	12.3%
	5=Strong preference global	2	1.3%

There was no significant association between learning style preference (active/reflective) and variables such as gender, study hours, study method, or study pattern (p>0.05). Overall, active vs. reflective learning appears independent of academic habits or demographics in this group (Table 2).

**Table 2:** Cross-tabulation of ‘Processing’ dimension of ILS (active/reflective) ILS: Index of Learning Styles

Variable	Group	Score=1	Score= 2	Score=3	Score=4	Score=5	Chi square, p value
Gender	Male	34 (32.7%)	5 (22.7%)	1 (33.3%)	6 (26.1%)	1 (50.0%)	1.44, 0.837
	Female	70 (67.3%)	17 (77.3%)	2 (66.7%)	17 (73.9%)	1 (50.0%)	
Study Hours	<1 hour	24 (23.1%)	3 (13.6%)	0 (0.0%)	6 (26.1%)	0 (0.0%)	4.46, 0.813
	1-3 hours	64 (61.5%)	14 (63.6%)	2 (66.7%)	13 (56.5%)	1 (50.0%)	
	>3 hours	16 (15.4%)	5 (22.7%)	1 (33.3%)	4 (17.4%)	1 (50.0%)	
Study Method	Group	6 (5.8%)	0 (0.0%)	0 (0.0%)	0 (0.0%)	0 (0.0%)	14.81, 0.063
	Individual	60 (57.7%)	9 (40.9%)	1 (33.3%)	20 (87.0%)	1 (50.0%)	
	Both	38 (36.5%)	13 (59.1%)	2 (66.7%)	3 (13.0%)	1 (50.0%)	
Study Pattern	Year-round	55 (52.9%)	15 (68.2%)	3 (100.0%)	11 (47.8%)	1 (50.0%)	4.68, 0.321
	Irregular	49 (47.1%)	7 (31.8%)	0 (0.0%)	12 (52.2%)	1 (50.0%)	

A statistically significant association was observed between study hours and sensing/intuitive preference with a p value of 0.03 meaning the distribution of three categories of study hours is not the same across all five categories of the ‘Perception’ dimension (Table 3).

**Table 3:** Cross-tabulation of ‘Perception’ dimension of ILS (sensing/intuitive) ILS: Index of Learning Styles

Variable	Group	Score=1	Score=2	Score=3	Score=4	Score=5	Chi square, p value
Gender	Male	27 (34.2%)	10 (20.0%)	2 (28.6%)	7 (41.2%)	1 (100.0%)	6.30, 0.177
	Female	52 (65.8%)	40 (80.0%)	5 (71.4%)	10 (58.8%)	0 (0.0%)	
Study Hours	<1 hour	21 (26.6%)	8 (16.0%)	1 (14.3%)	3 (17.6%)	0 (0.0%)	16.97, 0.03
	1-3 hours	48 (60.8%)	27 (54.0%)	5 (71.4%)	14 (82.4%)	0 (0.0%)	
	>3 hours	10 (12.7%)	15 (30.0%)	1 (14.3%)	0 (0.0%)	1 (100.0%)	
Study Method	Group	2 (2.5%)	4 (8.0%)	0 (0.0%)	0 (0.0%)	0 (0.0%)	9.10, 0.33
	Individual	50 (63.3%)	30 (60.0%)	3 (42.9%)	7 (41.2%)	1 (100.0%)	
	Both	27 (34.2%)	16 (32.0%)	4 (57.1%)	10 (58.8%)	0 (0.0%)	
Study Pattern	Year-round	45 (57.0%)	26 (52.0%)	3 (42.9%)	10 (58.8%)	1 (100.0%)	1.63, 0.802
	Irregular	34 (43.0%)	24 (48.0%)	4 (57.1%)	7 (41.2%)	0 (0.0%)	

No significant differences were observed in visual/verbal preferences across gender, study hours, study method, or pattern. The distribution of learning styles was evenly spread, and study behavior did not appear to influence visual/verbal orientation (Table 4).

**Table 4:** Cross-tabulation of the ‘Input’ dimension of ILS (visual/verbal) ILS: Index of Learning Styles

Variable	Group	Score=1	Score=2	Score=3	Score=4	Chi square, p value
Gender	Male	18 (40.0%)	18 (25.0%)	10 (29.4%)	1 (33.3%)	0.297, 0.396
	Female	27 (60.0%)	54 (75.0%)	24 (70.6%)	2 (66.7%)	
Study Hours	<1 hour	10 (22.2%)	19 (26.4%)	4 (11.8%)	0 (0.0%)	9.075, 0.169
	1-3 hours	26 (57.8%)	38 (52.8%)	27 (79.4%)	3 (100.0%)	
	>3 hours	9 (20.0%)	15 (20.8%)	3 (8.8%)	0 (0.0%)	
Study Method	Group	2 (4.4%)	3 (4.2%)	1 (2.9%)	0 (0.0%)	6.55, 0.364
	Individual	30 (66.7%)	45 (62.5%)	15 (44.1%)	1 (33.3%)	
	Both	13 (28.9%)	24 (33.3%)	18 (52.9%)	2 (66.7%)	
Study Pattern	Year-round	26 (57.8%)	38 (52.8%)	18 (52.9%)	3 (100.0%)	2.79, 0.424
	Irregular	19 (42.2%)	34 (47.2%)	16 (47.1%)	0 (0.0%)	

Significant associations were seen between gender (p=0.006), study pattern (p=0.039) and the sequential-global axis. Male students showed a higher global learning preference, while irregular study patterns were associated with intuitive styles (Table 5).

**Table 5:** Cross-tabulation of ‘Comprehension’ dimension of ILS (sequential/global) ILS: Index of Learning Styles

Variable	Group	Score=1	Score=2	Score=3	Score=4	Score=5	Chi-square, p value
Gender	Male	23 (23.0%)	9 (30.0%)	2 (66.7%)	12 (63.2%)	1 (50.0%)	14.42, 0.006
	Female	77 (77.0%)	21 (70.0%)	1 (33.3%)	7 (36.8%)	1 (50.0%)	
Study Hours	<1 hour	22 (22.0%)	5 (16.7%)	0 (0.0%)	5 (26.3%)	1 (50.0%)	10.44, 0.236
	1-3 hours	55 (55.0%)	21 (70.0%)	3 (100.0%)	14 (73.7%)	1 (50.0%)	
	>3 hours	23 (23.0%)	4 (13.3%)	0 (0.0%)	0 (0.0%)	0 (0.0%)	
Study Method	Group	4 (4.0%)	1 (3.3%)	0 (0.0%)	1 (5.3%)	0 (0.0%)	3.32, 0.913
	Individual	61 (61.0%)	16 (53.3%)	3 (100.0%)	10 (52.6%)	1 (50.0%)	
	Both	35 (35.0%)	13 (43.3%)	0 (0.0%)	8 (42.1%)	1 (50.0%)	
Study Pattern	Year-round	51 (51.0%)	22 (73.3%)	3 (100.0%)	9 (47.4%)	0 (0.0%)	10.07, 0.039
	Irregular	49 (49.0%)	8 (26.7%)	0 (0.0%)	10 (52.6%)	2 (100.0%)	

In this study, gender shows a significant association (p=0.03) with academic performance, with male students scoring slightly better than female students. The study pattern also showed a statistically significant effect on academic performance, with p=0.006, which can be interpreted that students following a year-round study pattern scored higher marks. But study hours, study method and all four learning style dimensions of ILS showed no significant impact on marks (Table 6).

**Table 6:** Association of categorical variables with academic performance

Variable	Group (code)	Mean Marks±SD	F-value	p-value
Gender	Male (1)	38.6383±11.0048	4.7637	0.0306
	Female (2)	35.0654±8.5399		
Tukey highly significant difference (HSD) post-hoc test: The means of the male female pair are significantly different. Male vs female: Diff=-3.5729, 95%CI=-6.8069 to -0.3389, p=0.0306				
Study Hours	<1 hour (1)	34.1818±9.3723	2.0551	0.1317
	1-3 hours (2)	37.383±9.6108		
	>3 hours (3)	34.2963±8.6683		
Study Method	Group (1)	27.8333±5.2694	2.4781	0.0873
	Individual (2)	36.6154±9.3223		
	Both (3)	36.2982±9.7486		
Study Pattern	Year-round (1)	38.0471±9.3934	7.9095	0.005567
	Irregular (2)	33.8261 ±9.0974		
Tukey HSD post-hoc test: Year round study Group vs irregular study Group: Diff=-4.2210, 95%CI=-7.1860 to -1.2560, p=0.0056				
Scale 1	Fairly balanced (1)	35.2885 ±9.7539	0.9466	0.4389
	Mod preference active (2)	37.9545 ±8.2778		
	Strong preference active (3)	33.3333±7.6376		
	Mod preference reflective (4)	38.8261±9.4178		
	Strong preference reflective (5)	35±8.4853		
Scale 2	Fairly balanced (1)	36.07±9.406	0.1382	0.9371
	Mod preference sensing (2)	36.11±7628		
	Strong preference sensing (3)	36.06±9.1782		
	Mod preference intuitive (4)	39.67±10.9697		
Scale 3	Fairly balanced (1)	36.07±9.406	0.1382	0.9371
	Mod preference visual (2)	36.11±9.7628		
	Strong preference visual (3)	36.0588±9.1782		
	Mod preference verbal (4)	39.6667±10.9697		
Scale 4	Fairly balanced (1)	35.59±8.7503	2.1769	0.0743
	Mod preference sequential (2)	38.5667±9.9054		
	Strong preference sequential (3)	44.6667±9.0738		
	Mod preference global (4)	35.3158±10.4086		
	Strong preference global (5)	23.5±20.5061		

## Discussion

Similar to the present study results, a study by Taheri et al. (2021) also showed that there was no significant relationship between the learning styles of students and their academic achievement [28]. In a study by Muniyapillai et al. (2023) also, no significant association was seen between the learning style and academic performance [29]. Similarly, studies conducted in India by Urvalet al. (2014), Mozaffari et al. (2020) in Iran, Mlambo (2011) in the West Indies, Samarakoon et al. (2013) in Sri Lanka and Liew et al.(2015) in Malaysia show a negative association between the learning style and academic performance [30-34]. Our results also tally with a study conducted by Ezanno et al. (2024) and another study at Queen’s University Belfast on a cohort of first-year medical and dental students. This study found varied learning styles, but no effect on academic performance [35,25].

In contrast, El-Bishouty et al. (2019) investigated the application of the Felder and Silverman learning style model in designing online courses [10]. They developed an interactive course analyzer tool that helps teachers assess the level of support provided by their courses for different learning styles. The findings indicated that designing courses with specific learning styles in mind can enhance the learning outcomes for students with those particular styles. The results suggest that considering students’ learning styles in course design can enhance the learning outcomes for those specific styles. A study conducted by Metgud et al. (2021) and Akhlaghi et al. (2018) in India, Habibpour Sedani et al. (2016) in Iran, Almigbal (2015) in Saudi Arabia and Ilçin et al. (2018) in Turkey also showed a positive association between learning style and academic performance [35-40]. These disparities may be primarily because of variations in sample size and the categorization of academic performance grading systems.

In this study, male students scored slightly better than female students. A study by Saxena et al. (2024) also showed that gender differences influence performance on United States Medical Licensing Examination® Step 1 (USMLE) Step 1, while a study by Algarni (2024) showed that female students in the flipped learning group scored higher academic scores [41,42].

The present study found that students following a year-round study pattern scored higher marks. Empirical analyses on large-scale unobtrusive behavioural data by Cao et al. (2018) also demonstrated that academic performance is significantly correlated with orderliness [43]. The results of a study by Aljaffer et al. (2024) also support the available literature, indicating a correlation between study habits and high academic performance [44]. Another study by Hou et al. (2020) also showed that physical fitness, library usage, and the regularity of lifestyle are significant contributors to academic performance among Chinese medical and dental students [45].

This study had several strengths and limitations that are worth mentioning. The strength of this study is the use of validated questionnaire (ILS) and a good sample size. The limitations of this study include the recruitment of second year students early in their course from a single institution, participant selection using non-probabilistic sampling procedures, and considering academic performance in a single subject, which may limit the generalizability of the results. Due to the nature of the cross-sectional study, temporal relation cannot be ascertained. Also, since there are more components of learning than just the learning styles, addressing learning styles alone cannot provide a solution to all learning problems. We relied on self-reported measures of learning styles, which may not accurately capture the complexity of individual learning preferences. Qualitative research methods, such as interviews or focus groups, can provide in-depth insights into how students with different learning styles experience and engage with educational content. This approach can identify specific challenges and opportunities for enhancing the design and delivery of educational materials and support services.

This study characterized learning styles among medical students and examined their association with academic performance. The absence of a significant relationship suggests that students of varying learning styles can perform equally well and academic performance depends more on the long-term regular study pattern.

## Conclusions

This study concludes that consistent study habit is more influential on academic performance than inherent learning styles. In this study, gender also shows an association with academic performance, with male students scoring slightly better than female students. Male student showed a higher global learning preference, while irregular study patterns were associated with intuitive styles in the 'Comprehension' dimension of ILS.

The findings of this study can provide input to curriculum designers to decrease the mismatch between the common learning styles and teaching methods in medicine and can serve as a foundation for future research with larger cohorts, which could further validate the conclusions of our study. A longitudinal study can be done to see how learning style preferences may change as the medical student progresses and how it impacts his or her performance in individual subjects. Utilizing advanced statistical techniques like machine learning algorithms, patterns and relationships in extensive datasets of student performance and learning style assessments can be assessed. This approach can uncover new insights and predictive models to improve the design and delivery of educational content.
